# Parallel and four-step synthesis of natural-product-inspired scaffolds through modular assembly and divergent cyclization

**DOI:** 10.3762/bjoc.8.105

**Published:** 2012-06-22

**Authors:** Hiroki Oguri, Haruki Mizoguchi, Hideaki Oikawa, Aki Ishiyama, Masato Iwatsuki, Kazuhiko Otoguro, Satoshi Ōmura

**Affiliations:** 1 Division of Chemistry, Graduate School of Science, Hokkaido University, Sapporo, Kita-ku, Hokkaido 060-0810, Japan; 2 Research Center for Tropical Diseases, Kitasato Institute for Life Sciences, Kitasato University, 5-9-1 Shirokane, Minato-ku, Tokyo 108-8641, Japan; 3 Graduate School of Infection Control Sciences, Kitasato University, 5-9-1 Shirokane, Minato-ku, Tokyo 108-8641, Japan

**Keywords:** chemical diversity, divergent cyclization, indole alkaloids, modular assembly, rhodium-catalyzed cyclization–cycloaddition, skeletal and stereochemical diversity

## Abstract

By emulating the universal biosynthetic strategy, which employs modular assembly and divergent cyclizations, we have developed a four-step synthetic process to yield a collection of natural-product-inspired scaffolds. Modular assembly of building blocks onto a piperidine-based manifold **6**, having a carboxylic acid group, was achieved through Ugi condensation, *N*-acetoacetylation and diazotransfer, leading to cyclization precursors. The rhodium-catalyzed tandem cyclization and divergent cycloaddition gave rise to tetracyclic and hexacyclic scaffolds by the appropriate choice of dipolarophiles installed at modules 3 and 4. A different piperidine-based manifold **15** bearing an amino group was successfully applied to demonstrate the flexibility and scope of the unified four-step process for the generation of structural diversity in the fused scaffolds. Evaluation of in vitro antitrypanosomal activities of the collections and preliminary structure–activity relationship (SAR) studies were also undertaken.

## Introduction

**Figure 1:**
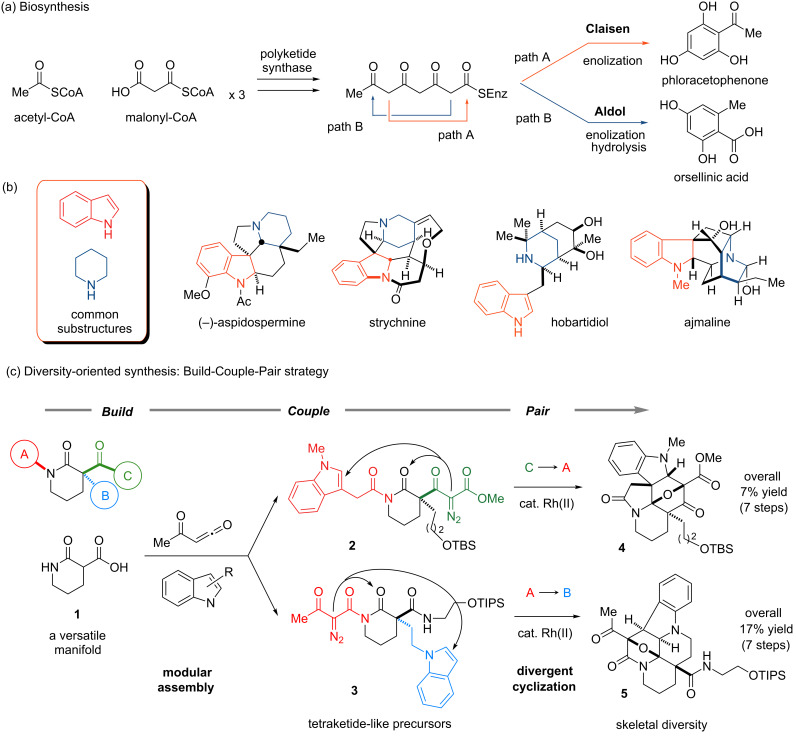
(a) Biosynthetic outline of aromatic polyketides; (b) structure of indole alkaloids composed of indole (red) and piperidine (blue) groups; (c) divergent cyclizations to generate scaffold variations as an illustration of the “Build-Couple-Pair” strategy in diversity-oriented synthesis.

Biologically intriguing natural products often possess cyclic scaffolds bearing dense arrays of functional groups and hydrogen-bond donors or acceptors. The incorporation of multiple sp^3^-centers on the scaffold creates a unique three-dimensional shape of the surface, which is responsible for specific molecular recognition with biomacromolecules in the cellular context [1–3]. To generate diverse collections of the elaborated cyclic scaffolds, nature has evolved biosynthetic machinery and often employs (1) modular assembly and (2) divergent cyclization [4]. As the simplest example of this structural diversification, the biosynthesis of aromatic polyketides is outlined in Figure 1a. Employing acetyl CoA as a starter unit, modular and iterative assembly of malonate extender units produces a linear tetraketide intermediate capable of being folded in at least two ways [5]. Intramolecular Claisen condensation and subsequent enolization produce phloracetophenone (path A), while aldol condensation followed by enolization and hydrolysis of the thioester yield orsellinic acid (path B). Inspired by this simple yet universal biosynthetic strategy, which generates structural variation among natural products, we envisioned the construction of chemical libraries featuring modular assembly for the rapid connection of simple building blocks, as well as divergent cyclization of a common precursor leading to distinct skeletons with complex molecular architectures.

Since the naturally occurring indole alkaloids share indole and piperidine as common substructures (Figure 1b) [6], we conceived the assembly of the substructures and subsequent intramolecular cyclization between these substructures to form the fused skeletons (Figure 1c). As a pioneering approach to shape the foundation of the “Build-Couple-Pair” (B/C/P) strategy [7–15] for diversity-oriented synthesis [16–17], a synthetic process to access indole-alkaloid-like scaffolds utilizing a piperidine-based manifold **1**, was developed in 2005 [18]. By exploiting lactam, carboxylic acid and β-ketocarbonyl functional groups on **1**, α-diazoketocarbonyl and indole groups were installed to produce a set of tetraketide-like precursors, **2** and **3**. Rhodium(II)-catalyzed tandem cyclization–cycloaddition [19–21] of the tetraketide-like precursors produced distinct multicyclic scaffolds, **4** and **5**, differing in the relative orientations of the substructures. This approach illustrates a systematic way of diversifying skeletal arrays in a controlled manner.

**Figure 2:**
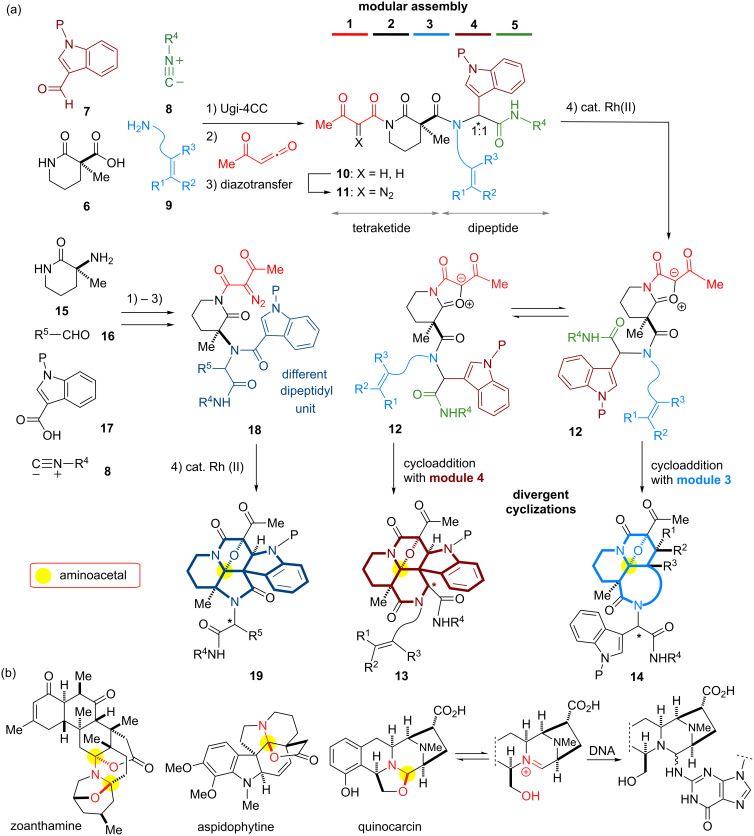
(a) Synthetic plans based on modular assembly and divergent cyclizations leading to fused skeletons; (b) structures of naturally occurring alkaloids bearing aminoacetal moieties and a proposed mode of action of quinocarcin.

With the intention to produce screening collections, we then devised a second-generation strategy applicable for a parallel synthetic protocol. This approach allows unified four-step access to a series of indole-alkaloid-like scaffolds. Some of these results were previously reported as a preliminary communication in 2009 [22]. As shown in Figure 2a, we conceived the modular assembly of three building blocks onto the piperidine-based manifold **6** with a carboxylic acid group. Ugi condensation [23–25] of **6** with indole-3-carbaldehyde **7**, isonitrile and amine building blocks **8** and **9**, followed by reaction with an acetylketene [26] would produce a tetraketide-like precursor **10** composed of five modules. Since two methylene groups in module 2 in the tetraketide-like moiety **10** are masked as an imide group and a quaternary center, respectively, the remaining methylene in module 1 would be regiospecifically manipulated through diazotransfer to form diazoimide **11** [27]. Rhodium(II)-catalyzed cyclization of **11** between modules 1 and 2 could generate a carbonium ylide intermediate **12**. In this system, there is a dynamic conformational equilibrium of the tertiary amide, which is expected to allow divergent cycloadditions with the dipolarophiles installed at modules 3 and 4 leading to either tetracyclic **14** or hexacyclic **13**. In this full account, we also employ a piperidine-based manifold **15** bearing an amino group in order to expand the applicability of the various building blocks in the four-step parallel synthesis. The modular assembly of **15** with **16**, **17** and **8** based on Ugi condensation could produce a different dipeptidyl array of the precursor **18**, which is expected to produce the distinct scaffold **19** compared to those produced from manifold **6**. According to this strategy employing rhodium(II)-catalyzed tandem reactions, four sp^2^-centers were efficiently converted into the corresponding sp^3^-centers, including an aminoacetal core. In nature, there are a variety of alkaloids that possess an aminoacetal group (Figure 2b). The aminoacetal groups embedded in the skeleton are prone to undergo C–O bond cleavage to form electrophilic iminium species, which allow covalent bond formation with biomacromolecules (nucleic acids, proteins) in a cellular environment, and thereby play pivotal roles in defining their biological activities [28–29]. As a mechanistic rationale for the antitumor activities of quinocarcins, DNA alkylation exploiting the iminium moiety was proposed as shown in Figure 2b [30].

Inspired by these biosynthetic strategies, we report herein the development of parallel and four-step synthetic processes, employing manifolds **6** and **15**, leading to collections of fused molecules with installations of diverse functional groups comprising aminoacetal, β-ketoimide and indole groups [31–34]. Evaluation of in vitro antitrypanosomal activities of the synthetic collections and preliminary SAR studies are also described [35–41].

## Results and Discussion

**Scheme 1:**
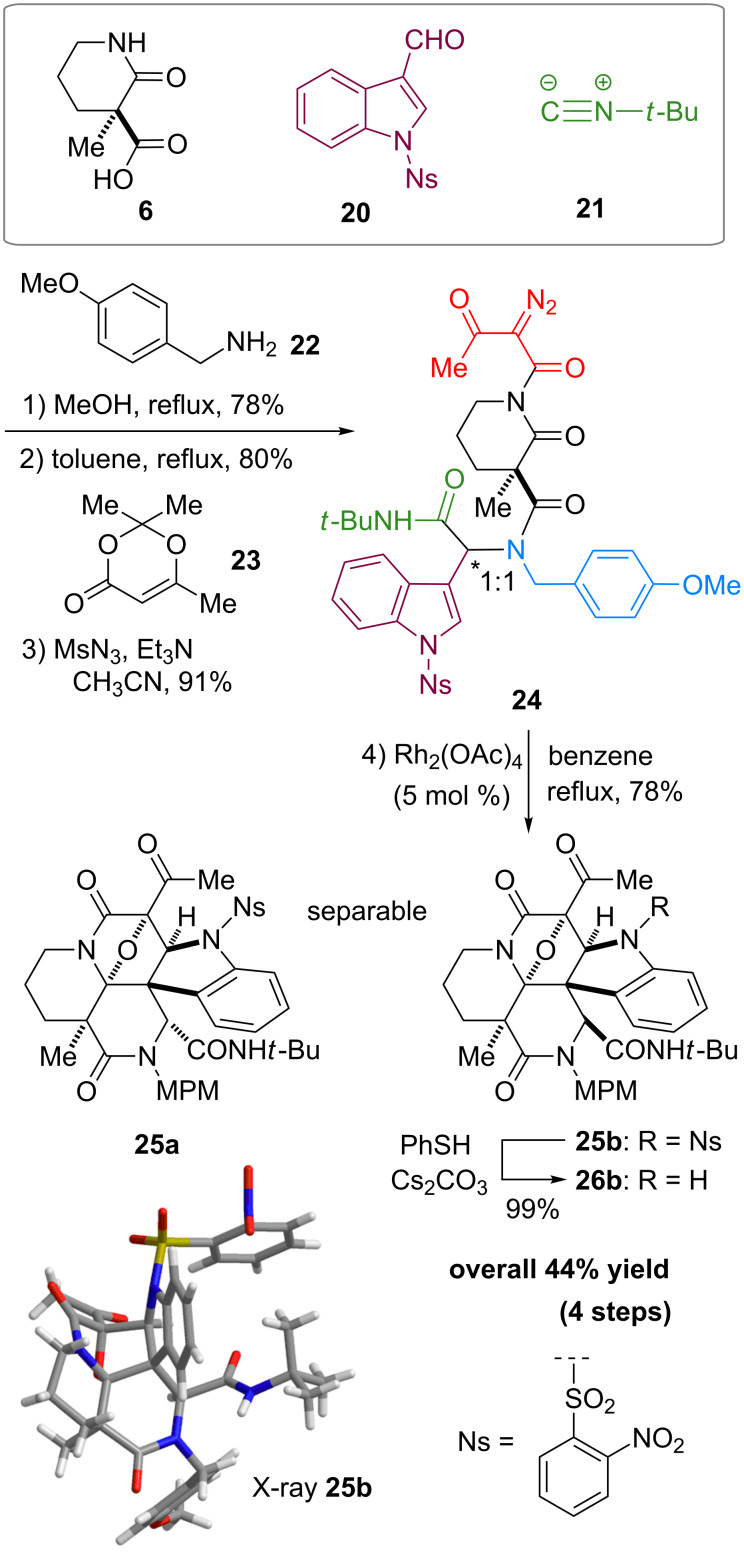
Four-step synthesis of hexacyclic skeleton **25**.

First, we assembled a linear precursor **24** with installation of a *p*-methoxybenzyl group and an indole ring at modules 3 and 4, respectively (Scheme 1), according to a procedure previously reported in our preliminary communication [22]. Racemic manifold **6**, indole-3-carbaldehyde derivative (**20**), *tert*-butylisonitrile (**21**) and *p*-methoxybenzylamine (**22**) were condensed in methanol under reflux to furnish a dipeptidyl product as a 1:1 diastereomeric mixture in 78% yield. *N*-Acetoacetylation of this intermediate was achieved by reaction with an acetylketene generated by heating of **23**. Subsequent diazotransfer reaction afforded the precursor **24** with a diazoimide group in 73% yield (two steps). Cyclization of **24** and subsequent cycloaddition between the resulting carbonium ylide and the indole C2–C3 double bond efficiently proceeded by the treatment with 5 mol % Rh_2_(OAc)_4_ catalyst in benzene under reflux to afford hexacyclic scaffold **25** in 78% yield. The cyclized products were obtained as a 1:1 diastereomeric mixture of **25a** and **25b** and were easily separable by conventional silica-gel chromatography. X-ray analysis of crystalline **25b** unambiguously determined the relative stereochemical relationships of the multiple sp^3^ centers embedded in the complex hexacyclic scaffold. In addition, removal of the *N*-nosyl protecting group by treatment with benzenethiol led to **26b** in quantitative yield [42].

**Scheme 2:**
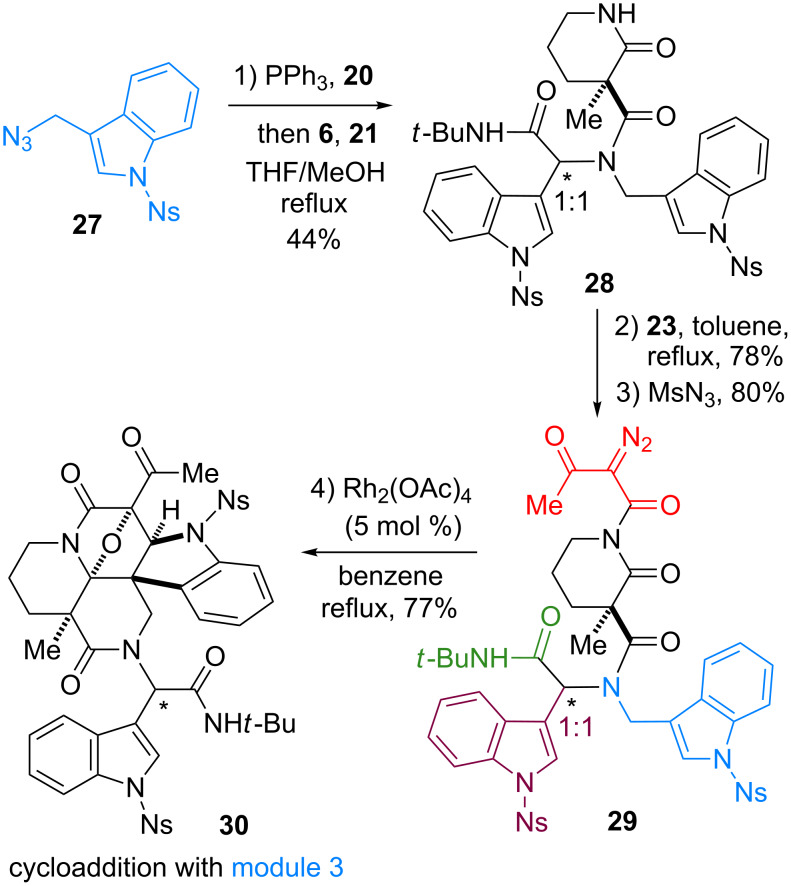
Four-step synthesis of hexacyclic skeleton **30**.

To generate skeletal variations by altering the sites of the cycloadditions, we next synthesized a branched precursor **29** bearing a pair of identical indole units at modules 3 and 4 (Scheme 2), as reported previously [22]. Due to the instability of the corresponding amine building block bearing the indole unit, azide **27** was employed as a precursor. Staudinger/aza-Wittig reaction [43] of **27** and **20** and subsequent condensation with **6** and **21** afforded the peptidyl product **28**. Installation of a β-keto imide followed by diazotransfer reaction produced **29**. Upon the treatment of **29** with Rh_2_(OAc)_4_ catalyst (5 mol %), the cycloaddition occurred in a highly site-selective manner at module 3 to form **30** in 77% yield. Cycloaddition with the other site (module 4) is likely to be hindered by the sterically demanding amide moiety (module 5) in the vicinity of the reaction centers.

**Scheme 3:**
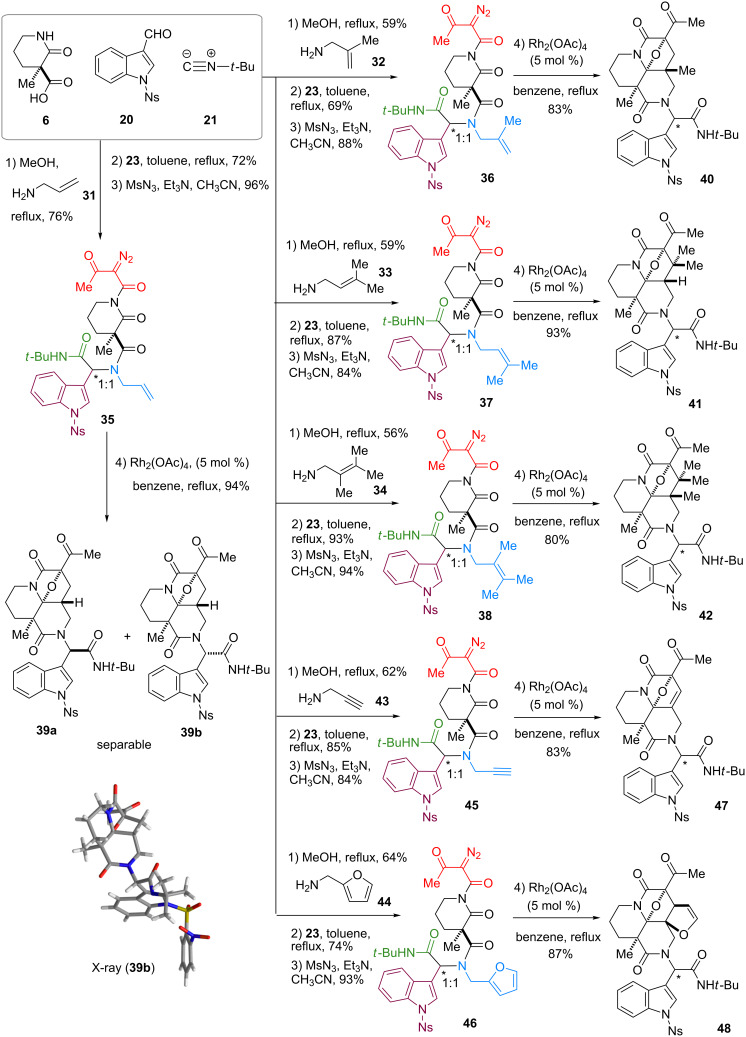
Parallel and four-step synthesis of tetracyclic skeletons **39**–**42** and **47**–**48**.

Taking into account the predominant involvement of the dipolarophile installed at module 3, we then designed a branched precursor **35** having a terminal olefin and an indole group at modules 3 and 4, respectively (Scheme 3). According to the previously reported protocol [22], Ugi reaction employing allylamine (**31**) and stepwise installation of a diazoimide group provided **35** in good yield. Upon treatment of **35** with Rh_2_(OAc)_4_ in benzene under reflux, 1,3-dipolar cycloaddition of the ylide intermediate with the terminal olefin at module 3 proceeded to give **39** as a separable 1:1 diastereomeric mixture in 94% yield. The relative stereochemistry of **39** was unambiguously determined by X-ray analysis of the crystalline **39b**.

In an effort not only to verify the reaction scope of the olefinic group installed at module 3 but also to shift the reaction site (module 3→4), we then prepared a series of cyclization precursors **36**–**38** in order of increasing steric hindrance of the olefinic groups as reported previously [22]. Allylamines **32**–**34** having a di-, tri- or tetra-substituted olefin were employed to synthesize precursors **36**–**38** based on the unified three-step protocol. The Rh(II)-catalyzed tandem cyclization–cycloaddition of the branched precursors **36**–**38** exclusively occurred at module 3. The cyclized products **40**–**42**, having the indole group at module 4 intact, were obtained in good yields. It is worth noting that the cycloadditions efficiently incorporated consecutive quaternary centers into the complex fused skeleton, overriding the considerable steric hindrance of the dipolarophiles composed of the tri- and even tetra-substituted olefin groups. To test the generality of the site-selective cycloaddition at module 3, we then synthesized precursors **45** and **46** with a terminal alkyne and a furan ring, respectively, by using amine building blocks **43** and **44** according to the reported procedure [22]. The Rh(II)-catalyzed tandem reactions of **45** and **46** again proceeded at module 3 to produce cyclized products **47** and **48** in good yields. Despite our concern for the potential instability of the aminoacetal moiety adjacent to the double bond, **47** is stable under the standard manipulations. Overall, the pair of diastereomers generated by the Ugi condensations were converted equally through the unified three-step transformations and easily separated after the cycloadditions.

**Scheme 4:**
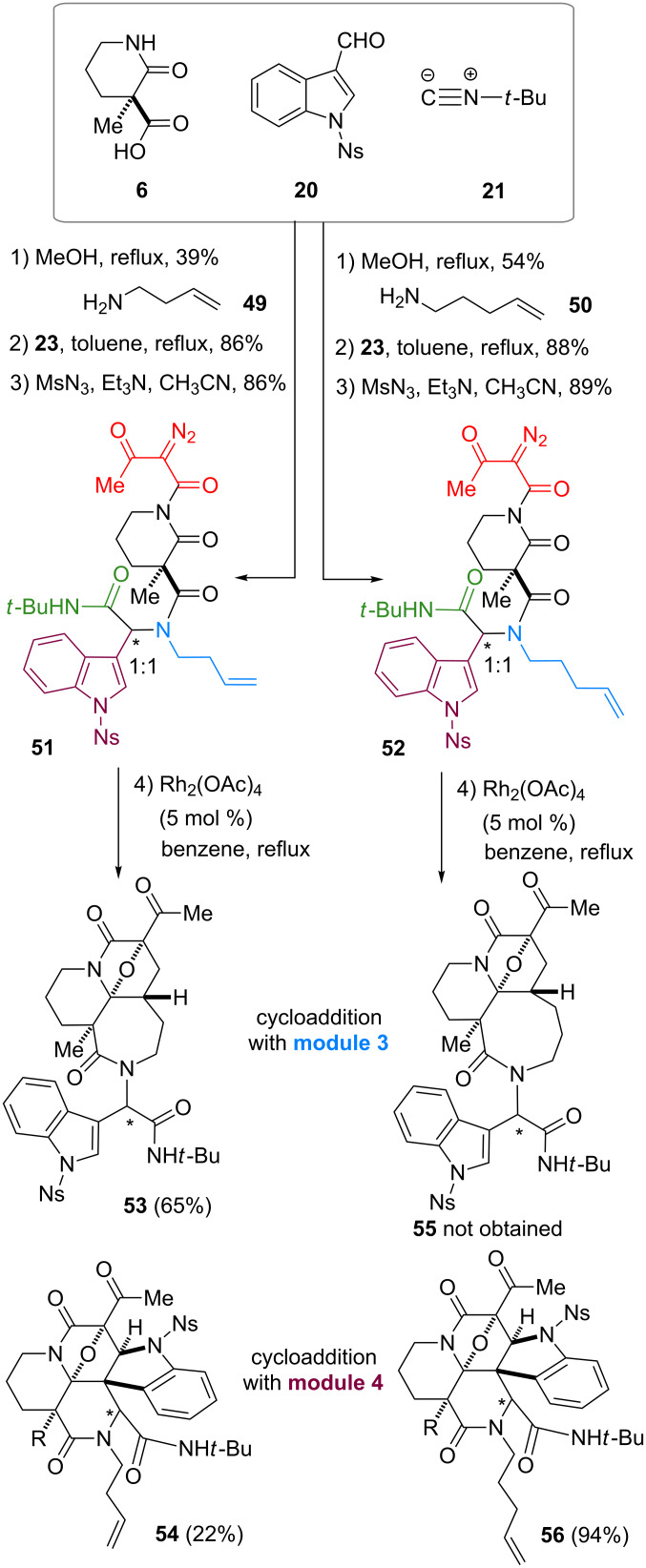
Synthesis of branched precursors, **51** and **52**, using amines **49** and **50**, with different methylene lengths and attempts to switch reaction sites.

Whilst the cycloadditions described above demonstrate the preference for the dipolarophile installed at module 3, we then attempted to alter the cyclization mode (module 3→4) by increasing the entropic barrier for medium-sized ring formation (Scheme 4) as reported previously [22]. For this purpose, we designed precursors **51** and **52**, synthesized through the three-step protocol employing amines **49** and **50**, respectively. Upon the treatment of **51** with Rh_2_(OAc)_4_, cycloaddition predominantly occurred at module 3 to produce tetracyclic **53** in 65% yield with formation of a seven-membered ring. Despite the minor pathway, cycloaddition at module 4 also competed to give **54** in 22% yield. On the other hand, cycloaddition of **52** exclusively occurred with the indole group at module 4, giving rise to **56** in 94% yield without eight-membered ring formation leading to **55**. X-ray analysis of the crystalline **56b** confirmed the structure [22]. Accordingly, alteration of the cyclization mode was achieved by modulating the ring sizes formed via cycloaddition, which allowed divergent access to hexacyclic and tetracyclic skeletons.

**Scheme 5:**
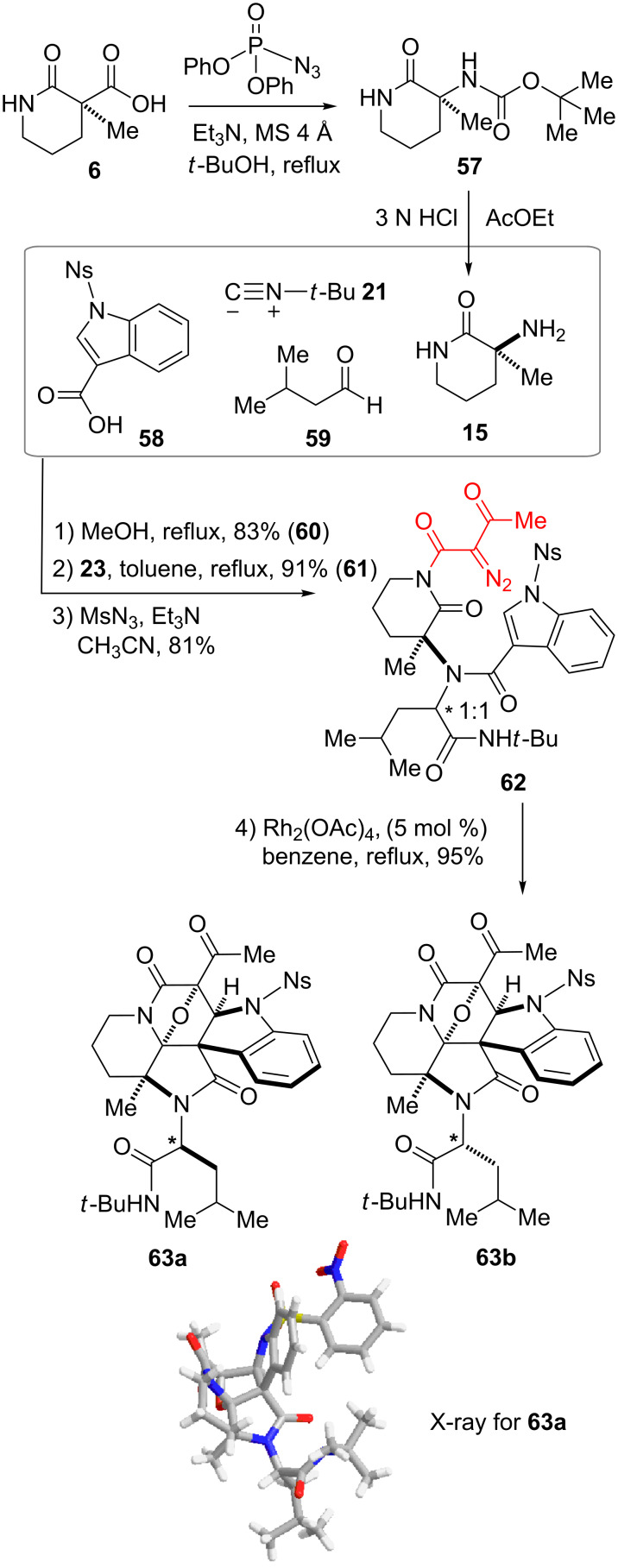
Four-step synthesis of hexacyclic scaffold **63** employing manifold **15**. For details of the synthesis of **60** and **61** see Supporting Information File 1.

In this study, we designed and synthesized a piperidine-based manifold **15** bearing an amino group in order to produce variations of branched precursors leading to distinct scaffolds (Scheme 5). The manifold **15** was readily prepared through Curtius rearrangement of **6** and subsequent removal of the resulting carbamate group. Ugi four-component condensation of **15**, isonitrile **21**, indole-3-carboxylic acid derivative **58** and aldehyde **59** produced a 1:1 diastereomeric mixture of the dipeptidyl intermediate. Stepwise installation of the α-diazocarbonyl group produced **62** in good yield. The cyclization precursor **62** has a different arrangement of the branched dipeptidyl unit linked to the piperidine-based manifold compared with those derived from **6**. Rhodium-catalyzed tandem cyclization–cycloaddition proceeded smoothly to produce **63** in 95% yield. After separation of the diastereomers, X-ray analysis of crystalline **63a** allowed its structural determination. The flexibility and divergence of the synthetic process with high levels of stereoselectivity are promising for the development of small-molecule libraries with structural diversity and complexity.

With collections of the natural-product-inspired molecules in hand, in vitro anti-trypanosomal activities [35–41] were evaluated by employing a GUTat 3.1 strain of *T. brucei brucei* (Table 1) according to the previously reported protocols (Supporting Information File 1). We found several hit compounds in the series of the cycloadducts exploiting module 3 as dipolarophiles. While compound **39a** shows negligible activities, the diastereomer **39b** exhibits the most potent activity (IC_50_ = 0.46 μg/mL), indicating the critical importance of the stereochemistry on the peptidyl unit (Table 1, entries 1 and 2). The IC_50_ value of the antitrypanosomal activity is comparable to or greater than those of the approved drugs, suramine and eflornithine. Unfortunately, **39b** exhibits relatively potent cytotoxicity (IC_50_ = 4.02 μg/mL) against a human cell line (MRC-5 cells), and its selectivity index (SI) is calculated to be 8.7 as a means to assess the combined potencies of both antitrypanosomal and cytotoxic activities. Incorporation of dimethyl substituents on the scaffold resulted in diminished activity (**41b**: IC_50_ = 5.89 μg/mL) (Table 1, entry 3). Removal of the nosyl group (**39b→39c**) also caused substantial loss of the activities, suggesting the critical role of the aromatic sulfone amide moiety (Table 1, entry 4). Aside from **25a**, which shows moderate activity (IC_50_ = 5.9 μg/mL) (Table 1, entry 5), the antitrypanosomal activities of hexacyclic compounds, **25b** and **30b**, (Table 1, entries 6 and 7) are negligible. In addition, the hexacycles (**63a** and **63b**) generated from manifold **15** also showed insignificant activities (data not shown). Thus, this preliminary assessment supports the idea that the collections of natural-product-inspired scaffolds could have high hit rates against biological screenings, even without having structural information about the biological targets and small-molecule modulators related to the targeted cellular functions. Further screening investigations of the synthetic collections prepared in the four-step process are currently underway in our laboratories.

**Table 1:** In vitro anti-trypanosomal activities of natural product analogues and approved drugs against *T. brucei brucei* GUTat 3.1^a^.

entry	compound	IC_50_ (μg/mL)		Selectivity Index (SI)
		anti-trypanosomal activity	cytotoxicity	

1	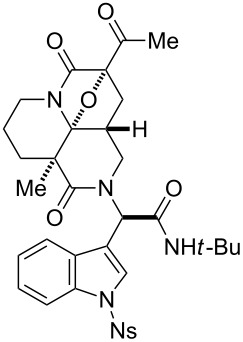 **39a**	>12.5	ND^b^	(─)
2	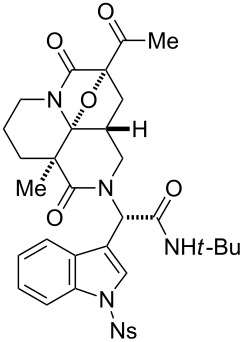 **39b**	0.46	4.02	8.7
3	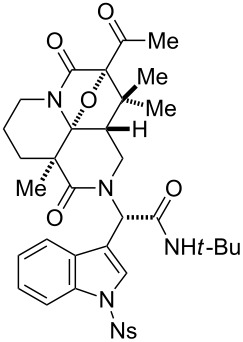 **41b**	5.89	34.64	5.9
4	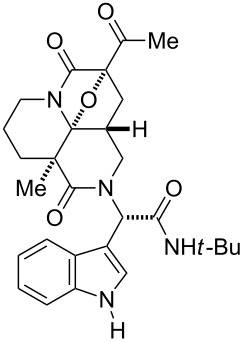 **39c**	>12.5	ND^b^	(─)
5	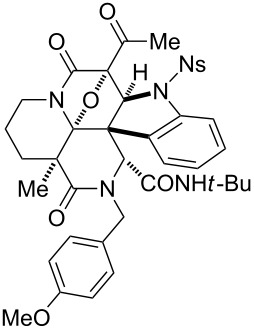 **25a**	5.9	24.47	4.1
6	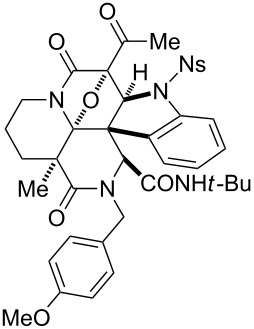 **25b**	>12.5	ND^b^	(─)
7	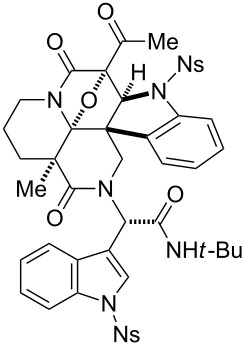 **30b**	>12.5	ND^b^	(─)
8	pentamidine^c^	0.00158	5.71	3600
9	suramin^c^	1.58	>100	>63
10	eflornithine^c^	2.27	>100	>44

^a^Culture of trypanosome (2.0–2.5 × 10^4^ trypanosomes/mL for GUTat 3.1 strain) was used. The cytotoxicities were evaluated with MRC-5 cells, and the selectivity index (SI) for trypanosomiasis was calculated as (IC_50_ for MRC-5)/(IC_50_ for *T. brucei brucei*). ^b^ND means “not determined”. ^c^Existing antitrypanosomal drugs.

## Conclusion

Inspired by biosynthetic strategies, we devised a modular assembly of five components employing manifold **6** and subsequent installation of a diazoimide group. This allowed three-step access to collections of cyclization precursors with a linkage of the piperidine and the indole units as key substructures shared with naturally occurring alkaloids. Rhodium-catalyzed cyclizations of diazoimides and subsequent divergent cycloadditions produced tetracyclic and hexacyclic scaffolds with exquisite regio- and stereocontrols. By the choice of dipolarophiles incorporated in modules 3 and 4, we have demonstrated site-selective cycloadditions leading to distinct scaffolds, which could be a rational approach to generate skeletal variations in synthetic collections. We further demonstrated the applicability of the manifold **15** bearing an amino group, which elicits further scaffold diversity. The parallel synthetic process based on the unified four-step sequences allows installation of dense arrays of various functional groups featuring aminoacetal, β-ketoimide and indole/olefin groups into multicyclic scaffolds reminiscent of natural products. Evaluation of antitrypanosomal activities of the collections allowed primary screenings of several hit compounds. The preliminary SAR study provided insights into the potential pharmacophore, based on the key features of scaffold, substructure and stereochemistry, which could be the proof of concept of our synthetic approach toward lead generation exploiting natural-product-inspired collections.

## Supporting Information

File 1 Experimental procedures and NMR spectra of compounds.
