# Supplementary material for: Parallel and four-step synthesis of natural-product-inspired scaffolds through modular assembly and divergent cyclization
Source: Beilstein J Org Chem. 2012 Jun 22;8:930–40. doi: 10.3762/bjoc.8.105 (PMC3388883; doi:10.3762/bjoc.8.105)
Supplement: File 1 — Experimental procedures and NMR spectra of compounds. [file Beilstein_J_Org_Chem-08-930-s001.pdf]

Supporting Information

for

Parallel and four-step synthesis of natural-product-inspired scaffolds through modular assembly and divergent cyclization

Hiroki Oguri*[1], Haruki Mizoguchi[1], Hideaki Oikawa[1], Aki Ishiyama[2], Masato Iwatsuki[2], Kazuhiko Otoguro[2] and Satoshi Ōmura[3]

Address: [1]Division of Chemistry, Graduate School of Science, Hokkaido University, Sapporo, Kita-ku, Hokkaido 060-0810, Japan, [2]Research Center for Tropical Diseases, Kitasato Institute for Life Sciences, Kitasato University, 5-9-1 Shirokane, Minato-ku, Tokyo 108-8641, Japan and [3]Graduate School of Infection Control Sciences, Kitasato University, 5-9-1 Shirokane, Minato-ku, Tokyo 108-8641, Japan

Email: Hiroki Oguri - oguri@sci.hokudai.ac.jp

* Corresponding author

Experimental procedures and NMR spectra of compounds

Table of contents

Experimental Section S2–S6

^{1}H and ^{13}C NMR Spectra S7–S10

General: NMR spectra were recorded on JEOL JNM-ECX 400 (^{1}H/400 MHz, ^{13}C/100 MHz) and JNM-ECX 600 (^{1}H/600 MHz, ^{13}C/150 MHz) spectrometers. Chemical shifts are reported in δ (ppm) using chloroform as an internal standard at δ 7.26 and 77.16 for ^{1}H and ^{13}C NMR, respectively. IR spectra were recorded on a JASCO FT/IR 660 Plus infrared spectrometer. Mass spectra were recorded on JEOL JMS-T100CS (ESI) spectrometer. The medium-pressure liquid chromatography (MPLC) purifications were performed on a YAMAZEN YFLC-AI-580. Where necessary, solvents were distilled from appropriate drying agents prior to use. Flash column chromatography was performed using Kanto Silica Gel 60N.

*Synthesis of manifold **15**:*

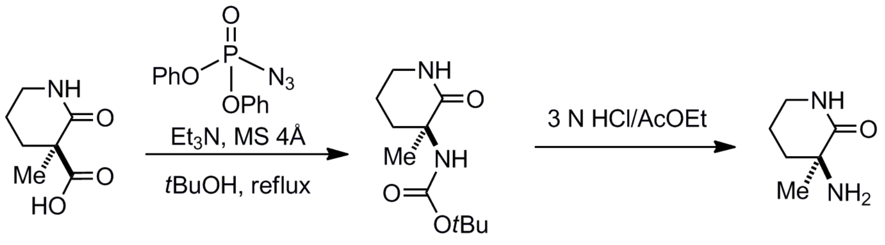

To a solution of **6** (715 mg, 4.55 mmol) in *t*-BuOH (22.8 mL) with molecular sieves 4 Å, diphenylphosphoryl azide (1.47 mL, 6.83 mmol) and Et_3N (1.14 mL, 8.19 mmol) were added at room temperature. After stirring at room temperature for 50 minute and heating under reflux for 24 h, the mixture was cooled to room temperature and filtrated through Celite®. To the filtrate was added water, and the mixture was extracted with EtOAc. The combined organic extracts were washed with 1 N HCl (aq), water, saturated $NaHCO_3$ (aq) and brine, dried over Na_2SO_4, filtrated through Celite®, and concentrated. The residue was purified by silica-gel column chromatography to afford **57** (795 mg, 3.48 mmol, 77%) as a white solid. Then, **57** (242 mg, 1.06 mmol) was treated with 3 N HCl/AcOEt (2 mL) at 0 °C and stirred overnight at room temperature. After being concentrated, the residue was basified with saturated $NaHCO_3$ (aq) and extracted with $CHCl_3$. Combined organic extracts were dried over Na_2SO_4 and concentrated to afford **15** (132 mg, 1.03 mmol, 98%) as a white solid.

*Four-step synthesis of **63**:*

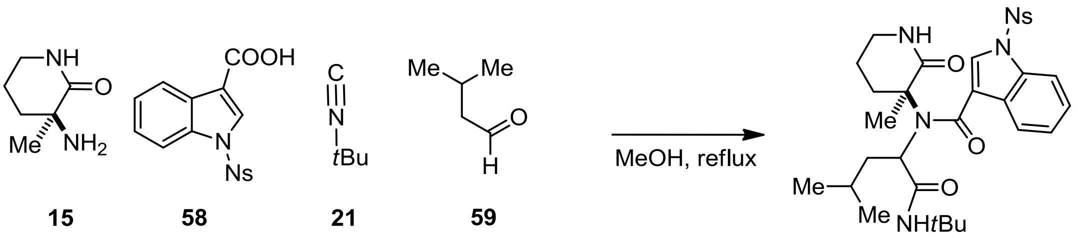

15 **58** **21** **59** **60**

To a suspension of amine **15** (58.0 mg, 0.453 mmol) and carboxylic acid **58** (173 mg, 0.500 mmol) in MeOH (2.5 mL), aldehyde **59** (97.0 μL, 0.901 mmol) and **21** (76.0 μL, 0.675 mmol) were added at room temperature. After heating under reflux for 6 h, the mixture was cooled to room temperature and concentrated. The residue was purified by silica-gel column chromatography to afford **60** (233 mg, 0.372 mmol, 83% for a 1:1 mixture of diastereomers) as a white solid.

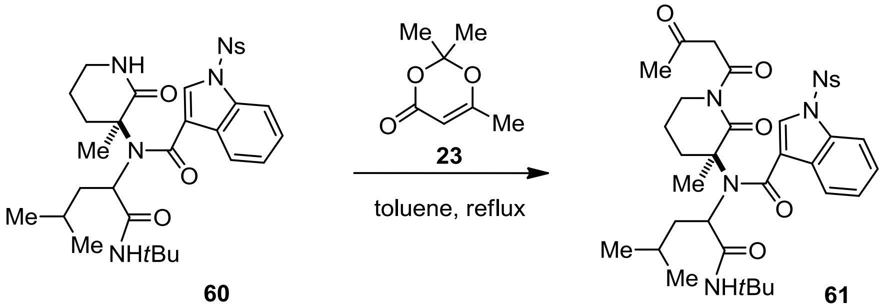

60 **61**

A solution of **60** (275 mg, 0.440 mmol) and **23** (117 μL, 0.880 mmol) in toluene (2.2 mL) was heated for 3.5 h under reflux. After being cooled to room temperature, the resulting mixture was concentrated and purified by silica-gel column chromatography to afford **61** (286 mg, 0.403 mmol, 91% for a 1:1 mixture of diastereomers) as a yellow amorphous solid.

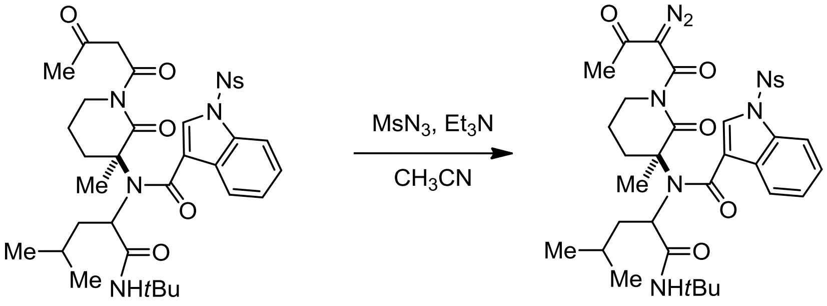

61 **62**

A solution of **61** (108 mg, 0.152 mmol) in acetonitrile (1.0 mL) was treated with Et_3N (63 μL, 0.451 mmol) and mesyl azide (26.0 μL, 0.308 mmol) and stirred for 3 h at room temperature. To the

mixture was added water, and it was then extracted with EtOAc. The combined organic extracts were washed with water and brine, dried over Na_2SO_4, and concentrated. The residue was purified by silica-gel column chromatography to afford **62** (88.9 mg, 0.121 mmol, 81% for a 1:1 mixture of diastereomers) as a pale yellow amorphous solid.

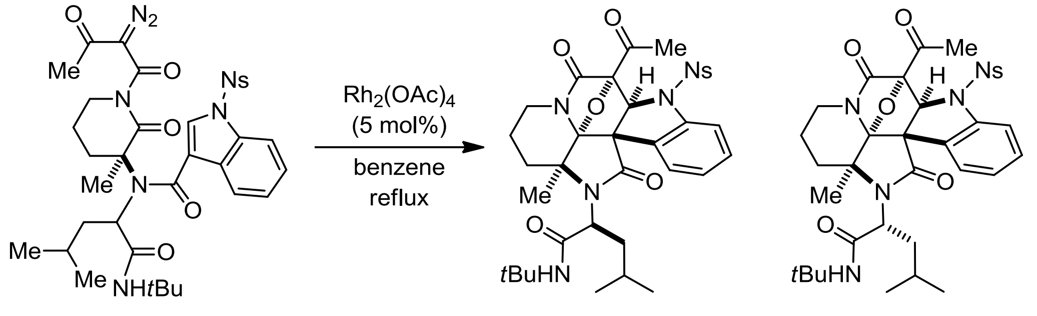

A solution of **62** (198 mg, 0.269 mmol) in benzene (3.0 mL) was treated with a catalytic amount of $Rh_2(OAc)_4 \cdot 2H_2O$ (6.7 mg, 5 mol %), and the mixture was heated for 2 h at 60 °C. The solution was cooled to room temperature and concentrated. The residue was purified by silica-gel column chromatography to afford **63** (181 mg, 0.255 mmol, 95% for a 1:1 mixture of diastereomers) as a white solid. These diastereomers were separable by silica-gel column chromatography (CH_2Cl_2/MeCN).

63a: ^{1}H NMR (600 MHz, $CDCl_3$) δ 0.99 (d, 3H, *J* = 6.5 Hz), 1.00 (d, 3H, *J* = 6.5 Hz), 1.29 (s, 9H), 1.54 (s, 3H), 1.60–1.71 (m, 2H), 1.80 (m, 1H), 1.88 (td, 1H, *J* = 13.4, 3.8 Hz), 2.12 (td, 1H, *J* = 14.1, 3.1 Hz), 2.37 (ddd, 1H, *J* = 13.4, 10.3, 4.8 Hz), 2.41 (br dt, 1H, *J* = 14.4, 2.8 Hz), 2.72 (s, 3H), 3.46 (dd, 1H, *J* = 13.1, 4.8 Hz), 3.92 (dd, 1H, *J* = 9.6, 5.2 Hz), 5.37 (s, 1H), 6.93 (s, 1H), 7.11 (dt, 1H, *J* = 7.6, 0.7 Hz), 7.30 (d, 1H, *J* = 7.6 Hz), 7.36 (ddd, 1H, *J* = 8.3, 7.6, 1.4 Hz), 7.42 (ddd, 1H, *J* = 7.9, 7.2, 1.7 Hz), 7.53 (dd, 1H, *J* = 7.9, 1.0 Hz), 7.55 (d, 1H, *J* = 8.3Hz), 7.60–7.65 (m, 2H); ^{13}C NMR (150 MHz, $CDCl_3$) δ 19.01, 19.91, 22.56, 22.61, 25.67, 28.61, 28.83, 33.39, 39.15, 39.26, 51.29, 60.32, 60.66, 69.33, 69.70, 90.85, 96.70, 118.08, 124.66, 125.20, 125.23, 125.67, 129.33, 129.84, 131.62, 131.98, 134.68, 143.52, 149.16, 165.37, 170.08, 170.71, 197.11; HRMS (ESI): $[M + Na]^+$ calcd for $C_{35}H_{41}N_5O_9SNa$, 730.2523; found, 730.2523.

63b: ^{1}H NMR (600 MHz, $CDCl_3$) δ 1.00 (d, 3H, *J* = 6.5 Hz), 1.01 (d, 3H, *J* = 6.5 Hz), 1.32 (s, 9H), 1.55 (s, 3H), 1.62 (m, 1H), 1.67 (sp, 1H, *J* = 6.5 Hz), 1.80–1.89 (m, 2H), 1.94 (td, 1H, *J* = 13.1, 3.4 Hz), 2.03 (dt, 1H, *J* = 13.4, 3.1 Hz), 2.18 (ddd, 1H, *J* = 13.7, 8.6, 6.9 Hz), 2.33 (br td, 1H, *J* = 13.4, 3.1 Hz), 2.66 (s, 3H), 3.49 (dd, 1H, *J* = 13.1, 4.5 Hz), 3.70 (t, 1H, *J* = 7.9 Hz), 5.35 (s, 1H), 7.16 (td, 1H, *J* = 7.6, 1.0 Hz), 7.17 (br s, 1H), 7.23 (dd, 1H, *J* = 7.6, 1.0 Hz), 7.40 (ddd, 1H, *J* = 8.3, 7.6, 1.4

Hz), 7.50 (ddd, 1H, J = 7.9, 7.6, 1.4 Hz), 7.57 (dd, 1H, J = 8.0, 1.0 Hz), 7.59 (d, 1H, J = 8.3Hz), 7.62 (dd, 1H, J = 7.9, 1.4 Hz), 7.66 (td, 1H, J = 7.6, 1.4 Hz); ^{13}C NMR (150 MHz, $CDCl_3$) δ 18.62, 19.81, 22.12, 23.28, 25.66, 28.63, 29.15, 34.22, 39.26, 39.39, 51.31, 59.26, 60.42, 69.09, 70.00, 91.38, 96.65, 117.89, 124.75, 125.04, 125.70, 126.34, 129.36, 129.51, 131.66, 131.84, 134.47, 143.37, 149.34, 165.35, 169.83, 170.34, 197.60; HRMS (ESI): $[M + Na]^+$ calcd for $C_{35}H_{41}N_5O_9SNa$, 730.2523; found, 730.2518.

The ^{1}H and ^{13}C NMR spectra of **63a** and **63b** are shown in Figure S1–S2 and S3–S4. The structure of **63a** was confirmed based on X-ray analysis. CIF file can be obtained free of charge from the Cambridge Crystallographic Data Center (CCDC): CCDC-870098.

Assay for in vitro anti-trypanosomal activity against *T. brucei* species

According to a procedure previously used in [Toriizuka et al. *Bioorg. Med. Chem.* **2008**, *16*, 10182–10189.], *Trypanosoma brucei brucei* strain GUTat 3.1 (Glasgow University, *Trypanozoon* antigenic type 3.1) was generously donated from Dr. Y. Yabu (Nagoya City University). *T. brucei brucei* GUTat 3.1 strain was cultured in IMDM with 3.024 g/L $NaHCO_3$, 100 μM hypoxanthine, 30 μM thymidine, 40 μM adenosine, 1.0 mM sodium pyruvate, 50 μM L-glutamine, 100 μM 2-mercaptoethanol, 50 U/mL of penicillin, and 50 μg/mL of streptomycin containing 10% heat-inactivated FBS at 37 °C, under 5.0% CO_2–95% air, according to the protocol of [Yabu et al. *Southeast Asian J. Trop. Med. Public Health* **1998**, *29*, 591]. In vitro antitrypanosomal activity of the test compounds was evaluated from a dose–response curve utilizing the fluorescent dye, Alamar Blue, according to the method of [Räz et al. *Acta Trop.* **1997**, *68*, 139 and Tasdemir et al. *Antimicrob. Agents Chemother.* **2006**, *50*, 1352] with some modifications. Ninety-five microliters of trypanosome suspension (2.0–2.5 x 10^4 trypanosomes/mL for GUTat 3.1 strain) of bloodstream forms was seeded into a 96-well microplate and 5.0 μL of test compound solution (dissolved in 5.0% dimethylsulfoxide: DMSO) was added. After incubating for 72 h at 37 °C under 5.0% CO_2–95% air, 10 μL of Alamar Blue was added to each well. After further incubation for 3–6 h at 37 °C under 5.0% CO_2–95% air, the microplate was measured at 528/20 nm excitation wavelength and 590/35 nm emission wavelength with an FLx800 fluorescent plate reader (Bio-Tek Instrument, Inc.Vermont, USA). Data were transferred into a graphic program (Excel) and the 50% inhibitory concentration (IC_{50}) values were determined with fluorescent plate reader software (KC-4, Bio-Tek).

Cytotoxicity tests on MRC-5 cells

The human diploid embryonic cell line, MRC-5, was a generous gift from Dr. L. Maes (Tibotec NV, Mechelen, Belgium). Cytotoxicity of the test compound was measured by the colorimetric MTT assay [Mossman, T. *J. Immunol. Methods* **1983**, *65*, 55 and Otoguro, K. et al. *ATLA:Alternatives to Laboratory Animals* **1991**, *19*, 352] in 96-well microplates. In brief, 100 μL of MRC-5 cell suspension was added to 96-well microplates at 1 × 10^3 cells/well and the cells were cultured for 24 h. Then, 90 μL of standard culture medium (MEM + 10% FCS) with or without 10 μL of test compound solution that was dissolved in 25% ethanol or 5% DMSO was added to each well. Further incubation was conducted at 37 °C under 5% CO_2–95% air for 7 days and then 20 μL of MTT–PBS solution (5 mg/mL) was added to each well. The microplate was incubated at 37 °C for 4 h under 5% CO_2–95% air. Then, the incubation medium was aspirated and 100 μL of DMSO was added to solubilize the MTT formazan product. After mixing, absorbance at 540 nm was measured with iEMS microplate reader MF. The IC_{50} value was estimated from the dose–response curve.

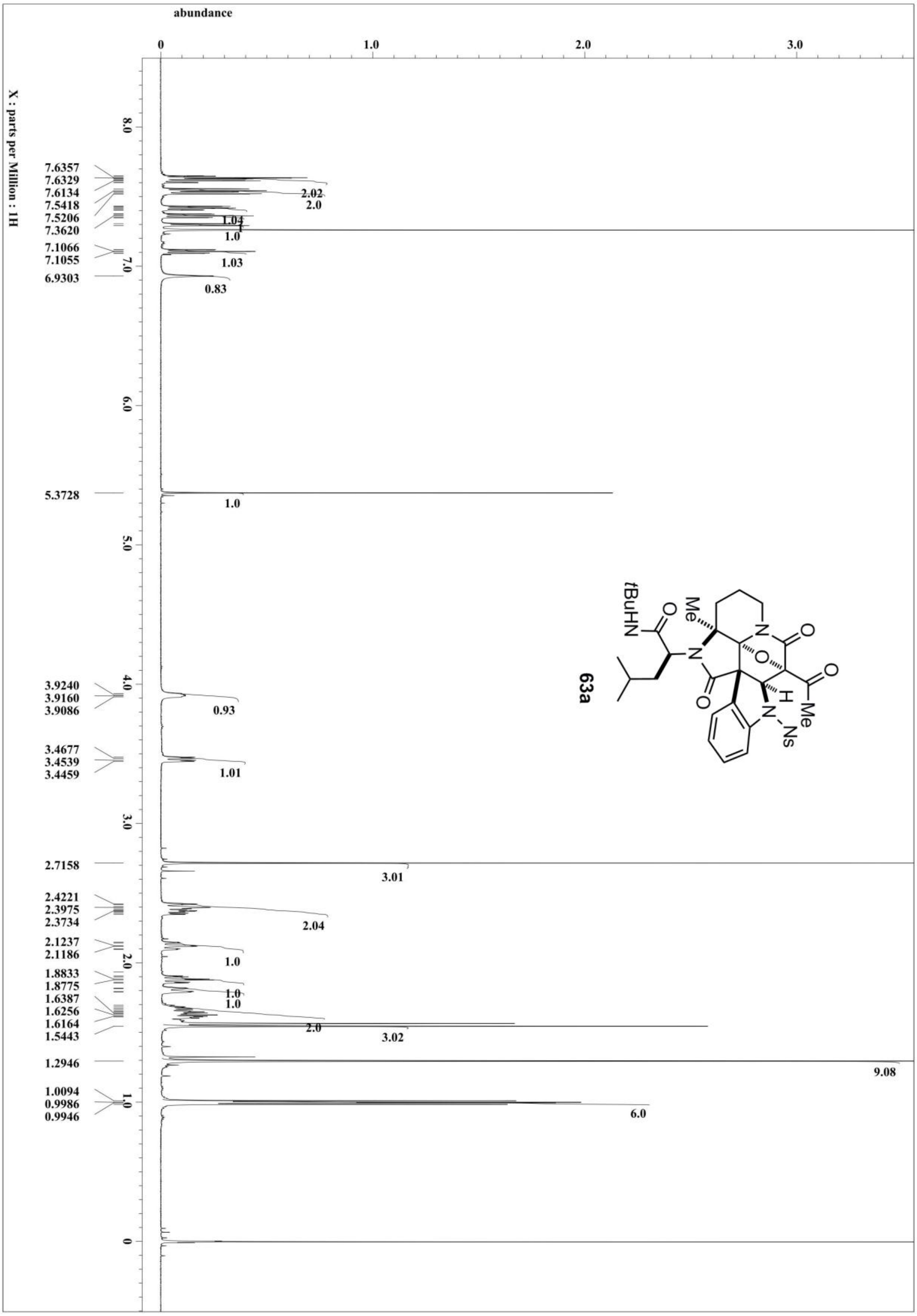

Figure S1: ^{1}H NMR spectrum of 63a in $CDCl_3$

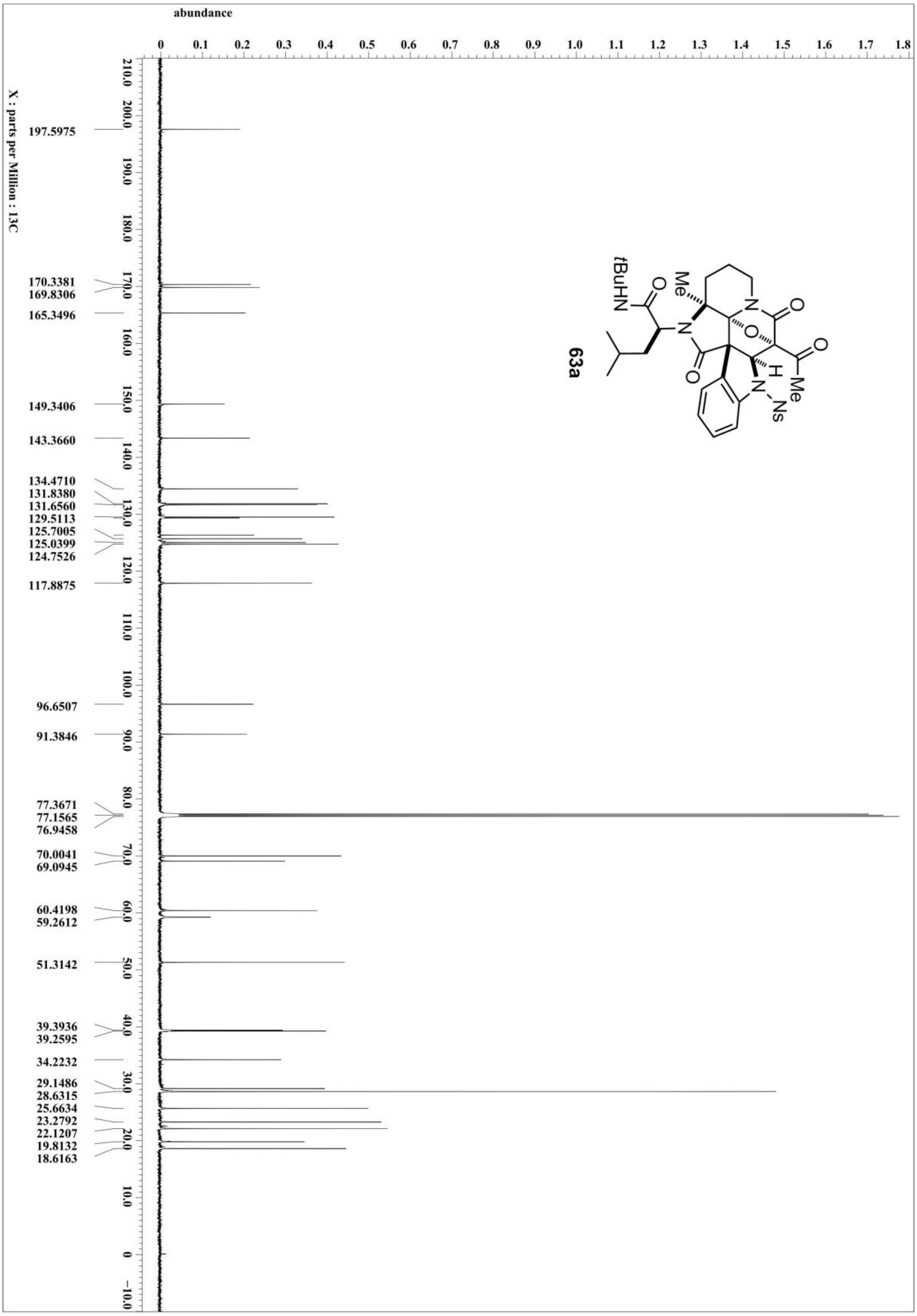

Figure S2: ^{13}C NMR spectrum of 63a in CDCl$_3$

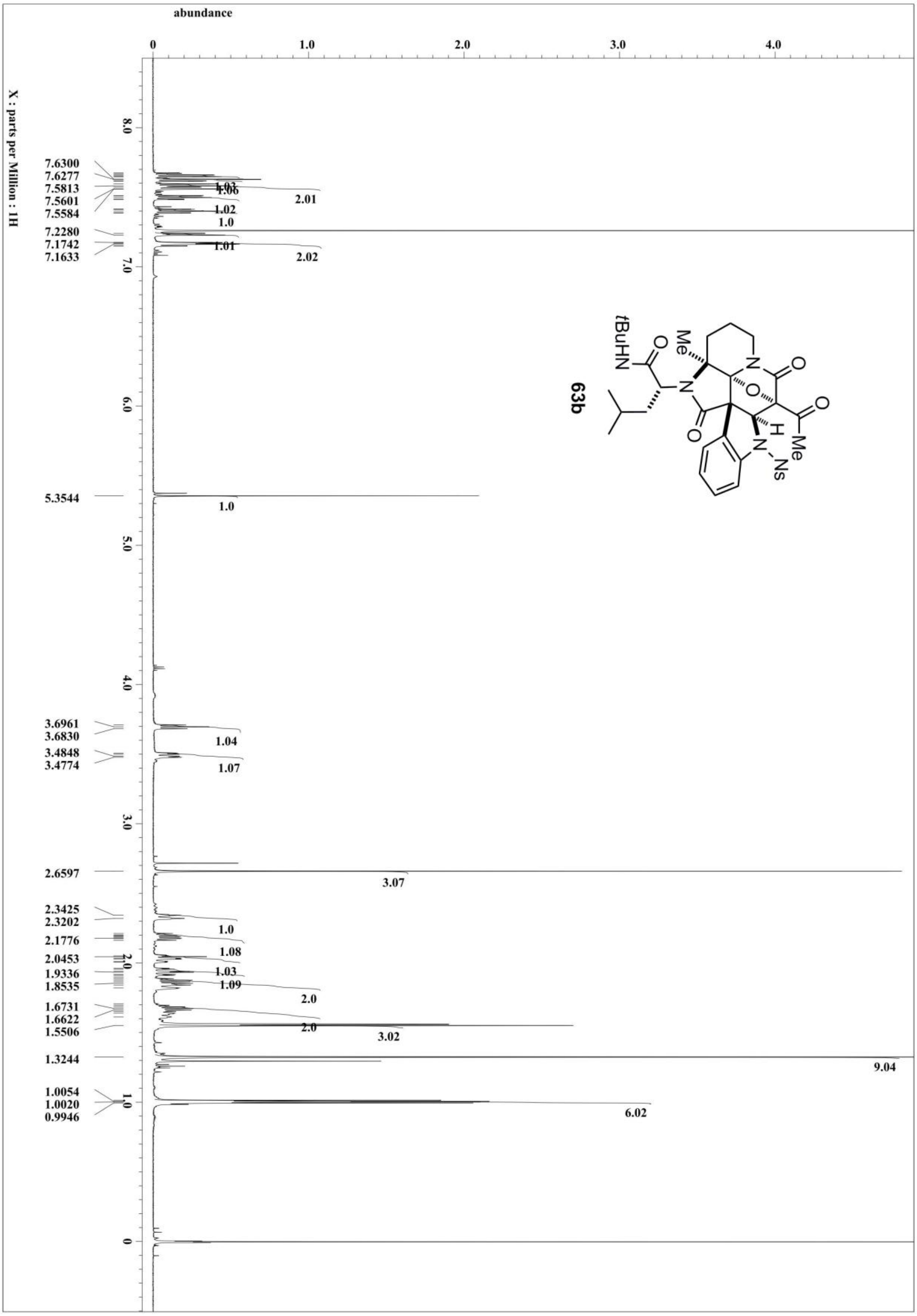

Figure S3: ^{1}H NMR spectrum of 63b in $CDCl_3$

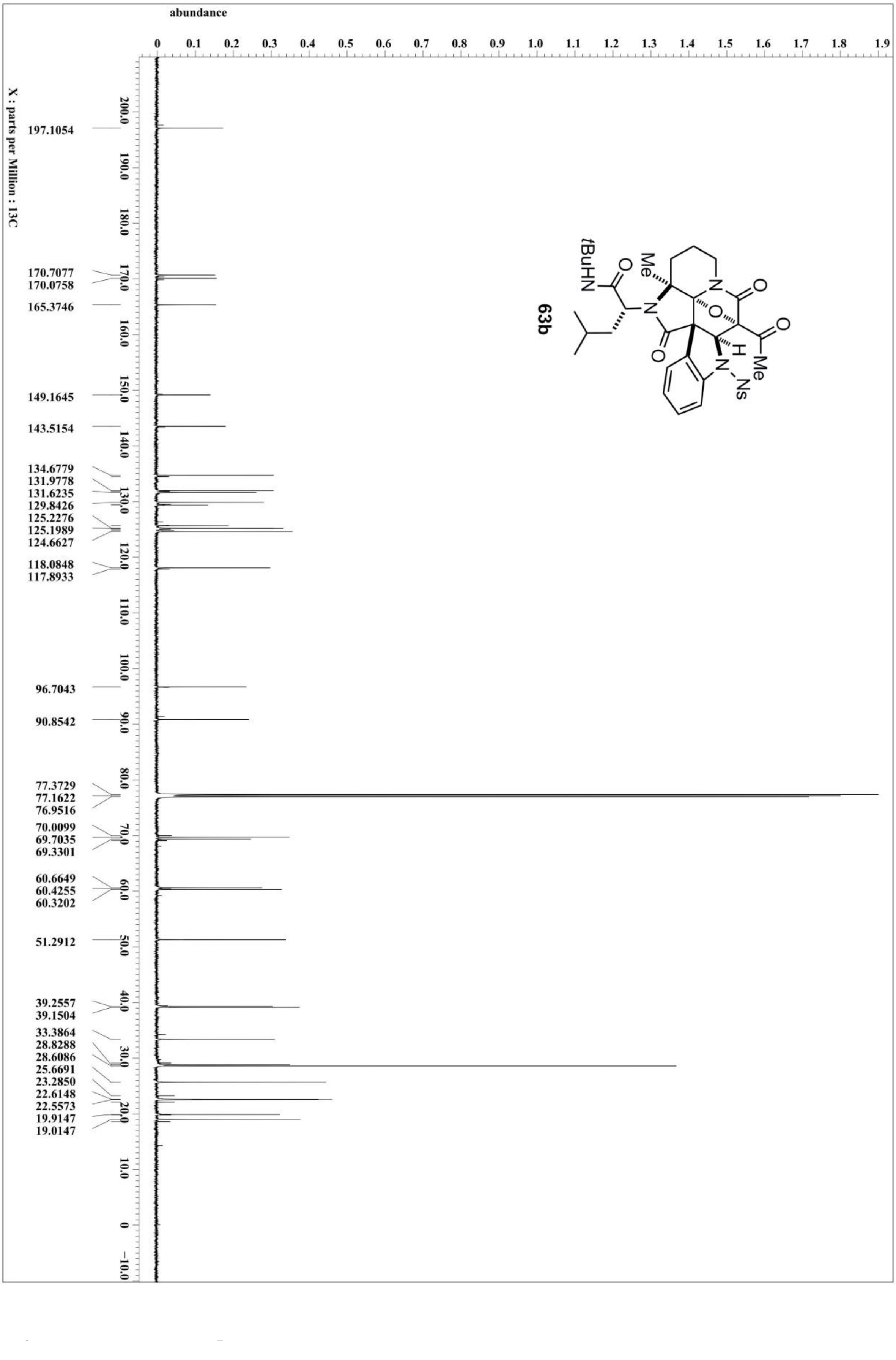

Figure S4: ^{13}C NMR spectrum of 63b in $CDCl_3$
